# Vitiligo in the Digital Spotlight: Retrospective Longitudinal Study in Germany

**DOI:** 10.2196/85171

**Published:** 2026-02-20

**Authors:** Enya Müller, Christine Gasteiger, Markus Böhm, Georg Pliszewski, Lilian Rauch, Tilo Biedermann, Alexander Zink

**Affiliations:** 1 Department of Dermatology and Allergy, Germany, School of Medicine and Health, Technical University of Munich, Biedersteiner Str.29, Munich, Germany, 49 8941403176; 2 Hautklinik, University of Münster, Münster, Germany; 3 Deutscher Vitiligo Bund, Adelsdorf, Germany

**Keywords:** vitiligo, chronic skin disease, pigmentary disorder, autoimmune skin disease, skin depigmentation, psychosocial burden, web search analysis, Google Ads Keyword Planner, digital epidemiology

## Abstract

**Background:**

Vitiligo is a chronic skin disease with a global prevalence of approximately 1% to 2%, characterized by depigmented macules. Little is known about the public interest and medical needs of patients with vitiligo in Germany. However, understanding this is critical for a patient-centered holistic therapeutic management of the disease.

**Objective:**

This study aimed to analyze vitiligo-related web search behavior across Germany as a proxy for public awareness. A retrospective longitudinal study was conducted using Google Ads Keyword Planner to collect monthly search volume data for vitiligo-related terms from October 2019 to May 2023.

**Methods:**

Keywords were identified in the 7 most spoken languages in Germany (German, Turkish, English, Arabic, Russian, and Polish). Seasonal and regional variations were analyzed, along with correlations with population density, dermatologist availability, and weather patterns.

**Results:**

In total, 7,764,080 vitiligo-related searches were recorded. Most searches (n=5,808,360, 74.81%) addressed general information. Search volume peaked during the summer months and correlated positively with temperature and sunshine hours (*P*<.001). Notable regional differences were observed, with the highest search rates in Hamburg, Berlin, and Bremen. Rural areas showed higher search volume per 100,000 inhabitants than urban areas.

**Conclusions:**

The findings suggest a strong public interest in vitiligo, particularly during periods of increased skin exposure. The high demand for treatment-related information further reflects the need for accessible, effective care. Web search behavior can offer real-time insights into public awareness and unmet needs, supporting earlier disease recognition, stigma reduction, and targeted educational strategies.

## Introduction

### Vitiligo

Vitiligo is a chronic skin disease with a global prevalence of approximately 1% to 2% and is characterized by depigmented round to oval macules [1]. These lesions may expand over time and develop on various body areas. The pigmentation of the skin and hair is primarily determined by melanin, a pigment synthesized by melanocytes. In individuals with vitiligo, melanocytes undergo degeneration or lose their function, leading to gradual loss of pigment cells. The underlying mechanisms of this process are multifactorial, involving genetic predisposition, immune-mediated pathways, and environmental influences. Vitiligo is associated with a substantial psychosocial burden, often resulting in emotional distress and social stigmatization [2,3]. Furthermore, patients with vitiligo exhibit an increased prevalence of autoimmune comorbidities, allergic conditions, and other dermatological diseases [4]. Recent studies have also suggested broader systemic implications, potentially linking vitiligo to dysregulations in immune homeostasis. Additionally, mental health concerns are frequently observed among affected individuals, with evidence indicating a higher prevalence of depression in this patient population [5,6]. A holistic therapeutic management of vitiligo includes shared decision-making with defined therapeutic goals that also integrates comorbidities and the impact of the disease on the patient’s quality of life. The currently available main therapeutic options are topical treatments (ruxolitinib, corticosteroids, and calcineurin inhibitors), systemic treatment (mainly oral corticosteroids), targeted and whole-body phototherapy surgical procedures (melanocyte transplantation), and depigmentation. Several new treatments are emerging [7,8]. Until now, a large proportion of patients with vitiligo appears to be unsatisfactorily treated, if not untreated [9,10]. Thus, seeking disease-related information beyond the typical medical settings, including on the internet, is common [11-14].

### Internet Data Mining

Search engine analyses have proven to be an effective method for quantifying public interest in health-related issues based on search volume data [15]. Understanding public interest in vitiligo-related topics provides valuable insights into patient concerns and informational needs about a disease that still has many unmet needs [8]. Given the widespread use of digital resources for health information, analyzing search trends offers a unique opportunity to identify common patient concerns, unmet medical needs, and knowledge gaps in dermatology. In Germany, approximately 95% of the population has internet access [16], with 90.2% preferring Google as their primary search engine. Additionally, 59% of internet users report seeking health-related information online during the last 12 months [17]. This widespread use of digital resources provides a valuable opportunity to gain a comprehensive overview of public interest and information-seeking behavior in dermatology through search engine analyses.

Search engine data have been previously used to explore various dermatological conditions, offering insights into public awareness, seasonal trends, and treatment preferences. Comparable analyses of more prevalent conditions such as atopic dermatitis, psoriasis, and pollen allergies have shown strong correlations between search volumes and real-world epidemiologic indicators, supporting the validity of web-based search data as a proxy for population-level disease interest even in rarer conditions such as vitiligo [18-21]. Studies have shown that search volume patterns reflect shifts in awareness, evolving medical needs, and the influence of environmental factors on disease perception [18,22-25]. In the case of vitiligo, global search engine analyses have demonstrated both an annual increase in search volume and seasonal fluctuations in a few countries, with peak interest observed during the summer months [12,13]. Cultural and media events can also shape public interest. For example, following the death of Michael Jackson, who publicly disclosed his vitiligo diagnosis, there was a marked increase in online searches related to the condition [12,17,26]. Additionally, geographic variations have been noted, with countries such as the United States that have a larger proportion of individuals with darker skin types exhibiting significantly higher search frequencies for vitiligo-related topics [13]. Against the backdrop of international research and the established role of web search analyses in identifying public health interests, our study investigated how vitiligo is reflected in German web search behavior. The aim of this study was to analyze internet search data on vitiligo across all of Germany in the 6 most widely spoken languages to evaluate public interest in the condition and examine temporal and regional variations in search behavior. By identifying patterns in online search activity, this study sought to highlight seasonal trends, geographic disparities, and areas of unmet informational needs.

## Methods

### Study Design

This retrospective longitudinal study analyzed Google search volumes for vitiligo-related terms in Germany between October 2019 and May 2023. While initially designed for online marketing, Google Ads Keyword Planner has been widely adopted in scientific research for analyzing public health interests. It was selected as the primary data source because it provides absolute monthly search volumes rather than relative indexes, allowing for quantitative assessment of population-level search behavior. Previous research has established its reliability and reproducibility for epidemiologic and dermatologic investigations in Germany and internationally [18,22,23,27-29]. Google Ads Keyword Planner operates by aggregating anonymized queries entered into the Google search engine and estimating their average monthly search volumes using proprietary algorithms based on Google’s advertising database. The tool identifies related search terms through semantic clustering and keyword co-occurrence analysis, providing quantitative data on search frequency stratified by geographic region, language, and time frame. Google Ads Keyword Planner was used to identify search terms (keywords) associated with vitiligo and extract the average monthly search volume. Search volume represents the number of user queries for specified terms. To ensure broad population coverage, the 6 most widely spoken languages in Germany (German, Turkish, English, Arabic, Russian, and Polish) were included. The analysis was performed using Google geotargeting, encompassing all 16 federal states and 1960 regional units across Germany. This analysis is based exclusively on data obtained from the Google search engine via Google Ads Keyword Planner. Searches on other engines (eg, Bing, Yahoo, or DuckDuckGo) were not included. This restriction was intentional as Google consistently accounts for more than 90% of all web searches in Germany [17], making it a highly representative proxy for overall internet search behavior in this context. For reasons of readability, the terms “internet search” or “web search” are occasionally used in the manuscript; however, all data reported are derived exclusively from the Google search engine.

This paper is based on a not yet published dissertation (C Gasteiger, unpublished data, 2025).

### Ethical Considerations

As the data used in this study were publicly available and anonymized, no ethics approval or informed consent was required.

### Categorization

Google Ads Keyword Planner was queried separately for each of the 6 most frequently spoken languages in Germany (German, English, Turkish, Arabic, Russian, and Polish) using the disease-related seed terms “vitiligo” and its direct translation or synonym in the respective language. For each language, the tool automatically generated a list of related keywords that users frequently entered in semantic or contextual relation to the seed term.

In an initial cleaning step, all duplicates and non–health-related entries were removed. The remaining keywords were categorized by 2 researchers (CG and AZ) according to thematic similarity and search intent. For the foreign languages (Turkish, Arabic, Russian, and Polish), machine translations (Google Translate) were first used to obtain English- and German-language equivalents. These translations were then verified and corrected by native speakers with a medical or linguistic background to ensure contextual accuracy and avoid cultural misinterpretations. To assess different areas of public interest, vitiligo-related keywords were categorized into 7 groups based on previous Google web search analyses and adapted to our dataset [12,13]: “general information,” “symptoms,” “affected body areas,” “psychosocial aspects,” “treatment options,” “vitiligo experts,” and “others.” After harmonizing the categories across all 6 languages, the entire keyword list was independently reviewed by 2 board-certified dermatologists (CG and AZ) experienced in vitiligo and digital epidemiology. Both experts independently rated each keyword as relevant or irrelevant to vitiligo based on clinical plausibility. Disagreements were resolved through discussion until consensus was reached.

The total search volume was calculated and descriptively analyzed.

Keywords in Arabic, Russian, Polish, and Turkish were translated into German and English using Google Translate and Linguee (DeepL SE). To minimize translation bias, native speakers reviewed and refined the translations where necessary to ensure accuracy and contextual relevance. To assess regional and seasonal differences in search behavior, the monthly search volume was calculated and expressed as search volume per 100,000 inhabitants. Districts with fewer than 150 inhabitants per square kilometer were classified as rural, whereas those exceeding this threshold were defined as urban [30]. Population density information was obtained from official German sources [31]. Dermatologist density per 100,000 inhabitants was obtained from the German National Association of Statutory Health Insurance Physicians [32]. German weather data (sunshine hours, precipitation, and average temperature) were obtained from the European Climate Assessment & Dataset project and standardized in R (R Foundation for Statistical Computing) by subtracting the mean and dividing by the SD [33]. For the analysis of seasonal trends, monthly search volumes were grouped into the 4 European seasons: spring (March-May), summer (June-August), autumn (September-November), and winter (December-February).

### Statistical Analysis

Statistical analyses were performed using Python (Python Software Foundation). Descriptive statistics were generated for all identified and categorized keywords. Seasonal variations in search volume were examined using the Kruskal-Wallis test, whereas regional differences between federal states were analyzed using the Friedman test. The Spearman correlation test was applied to assess associations between vitiligo-related search volumes and environmental as well as demographic factors, including weather conditions, dermatologist density, population density, pollen levels, and epidemiological data (COVID-19 and influenza cases). The significance threshold was set at *P*<.05. Nonparametric tests were chosen because the distribution of search volumes violated normality assumptions (Shapiro-Wilk test; *P*<.001). Therefore, the Kruskal-Wallis and Friedman tests were applied to account for nonnormally distributed and heteroscedastic data. To address potential temporal autocorrelation, the analyses were based on aggregated monthly data rather than daily values, reducing short-term dependency effects. Regional clustering was considered by comparing standardized search volumes per 100,000 inhabitants across federal states.

## Results

### Overview

**Figure 1.**
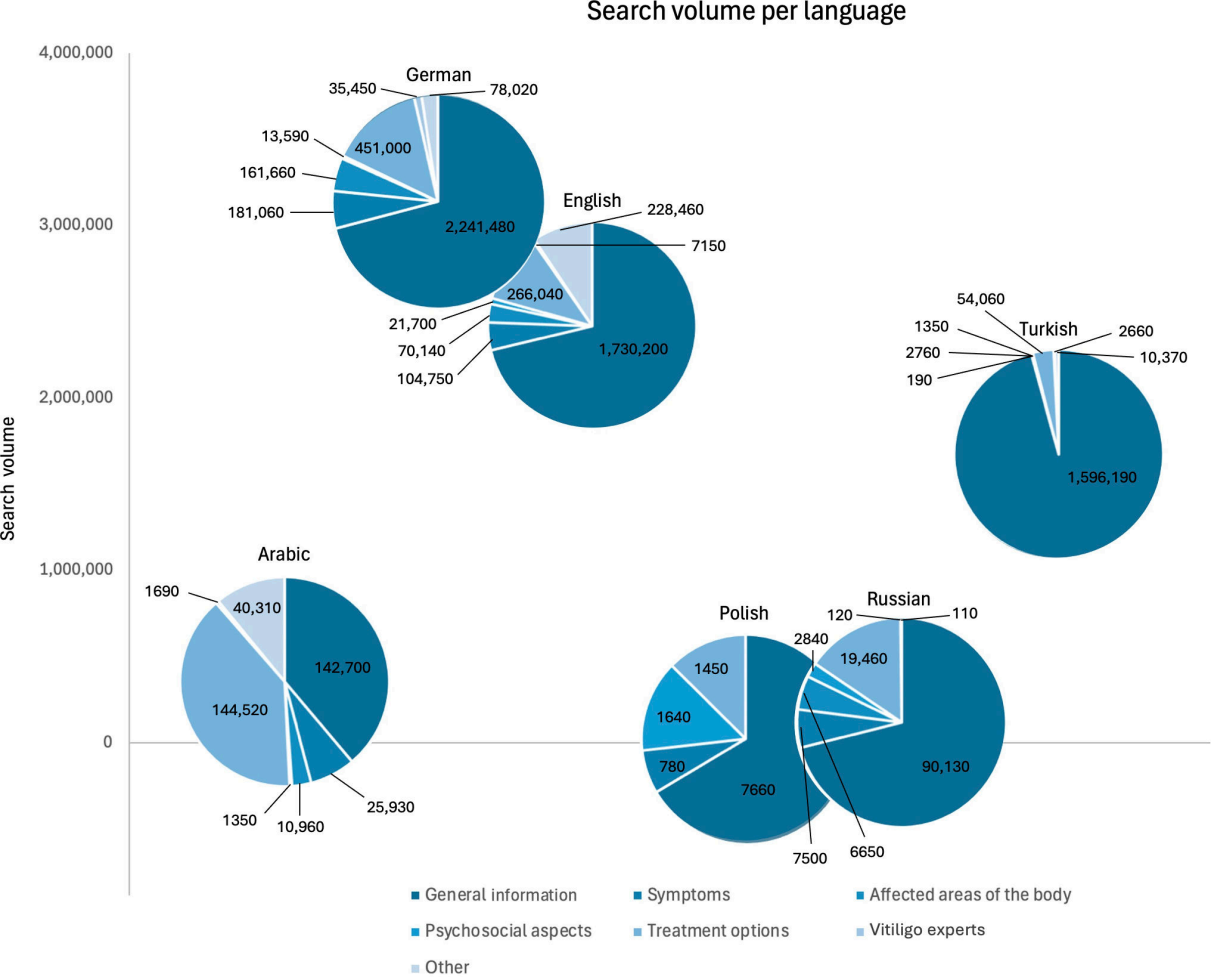
Search behavior for vitiligo: keyword frequency and search volume by language and category.

During the study period, a total of 7,764,080 searches related to vitiligo were found in the 6 most common languages in Germany. The highest proportion of searches were in German, with 40.73% (n=3,162,260) of the queries, followed by English (n=2,428,440, 31.28%) and Turkish (n=1,667,580, 21.48%; Table 1 and Figure 1). Google Ads identified the most keywords in Arabic (n=1074), followed by English (n=823). Polish had the lowest number of keywords at only 22 (Table 1).

**Table 1.** Comparison of the absolute and relative Google search volume of terms related to vitiligo in Germany from October 2019 to May 2023. Keywords are search terms entered by users into the Google search engine, representing real-world queries (n=7,764,080).

	Total	Arabic (n=367,460)	German (n=3,162,260)	English (n=2,428,440)	Polish (n=11,530)	Russian (n=126,810)	Turkish (n=1,667,580)
Number of keywords	2878	1074	284	823	22	265	410
Searches on “general information,” n (%)	5,808,360 (74.81)	142,700 (38.83)	2,241,480 (70.88)	1,730,200 (71.25)	7660 (66.44)	90,130 (71.07)	1,596,190 (95.72)
Searches on “symptoms,” n (%)	320,210 (4.12)	25,930 (7.06)	181,060 (5.73)	104,750 (4.31)	780 (6.76)	7500 (5.91)	190 (0.01)
Searches on “affected body areas,” n (%)	252,170 (3.25)	10,960 (2.98)	161,660 (5.11)	70,140 (2.89)	0 (0.00)	6650 (5.24)	2760 (0.17)
Searches on “psychosocial aspects,” n (%)	42,470 (0.55)	1350 (0.37)	13,590 (0.43)	21,700 (0.89)	1640 (14.22)	2840 (2.24)	1350 (0.08)
Searches on “treatment options,” n (%)	936,530 (12.06)	144,520 (39.33)	451,000 (14.26)	266,040 (10.96)	1450 (12.58)	19,460 (15.35)	54,060 (3.24)
Searches on “vitiligo experts,” n (%)	47,070 (0.61)	1690 (0.46)	35,450 (1.12)	7150 (0.29)	0 (0.00)	120 (0.09)	2660 (0.16)
Searches on “others,” n (%)	357,270 (4.60)	40,310 (10.97)	78,020 (2.47)	228,460 (9.41)	0 (0.00)	110 (0.09)	10,370 (0.62)

### Search Categories

Vitiligo-related search queries were divided into 7 categories (Table 1). The category with the highest search volume in all languages except Arabic was “general information about vitiligo.” In Arabic, “treatment options” was the most frequently searched category, accounting for 39.33% (144,520/367,460) of all Arabic-language queries. Across all other languages, “treatment options” consistently ranked as the second most frequently searched category (German: 451,000/3,162,260, 14.26%; English: 266,040/2,428,440, 10.96%; Polish: 1450/11,530, 12.58%; Russian: 19,460/126,810, 15.35%; Turkish: 54,060/1,667,580, 3.24%).

**Figure 2.**
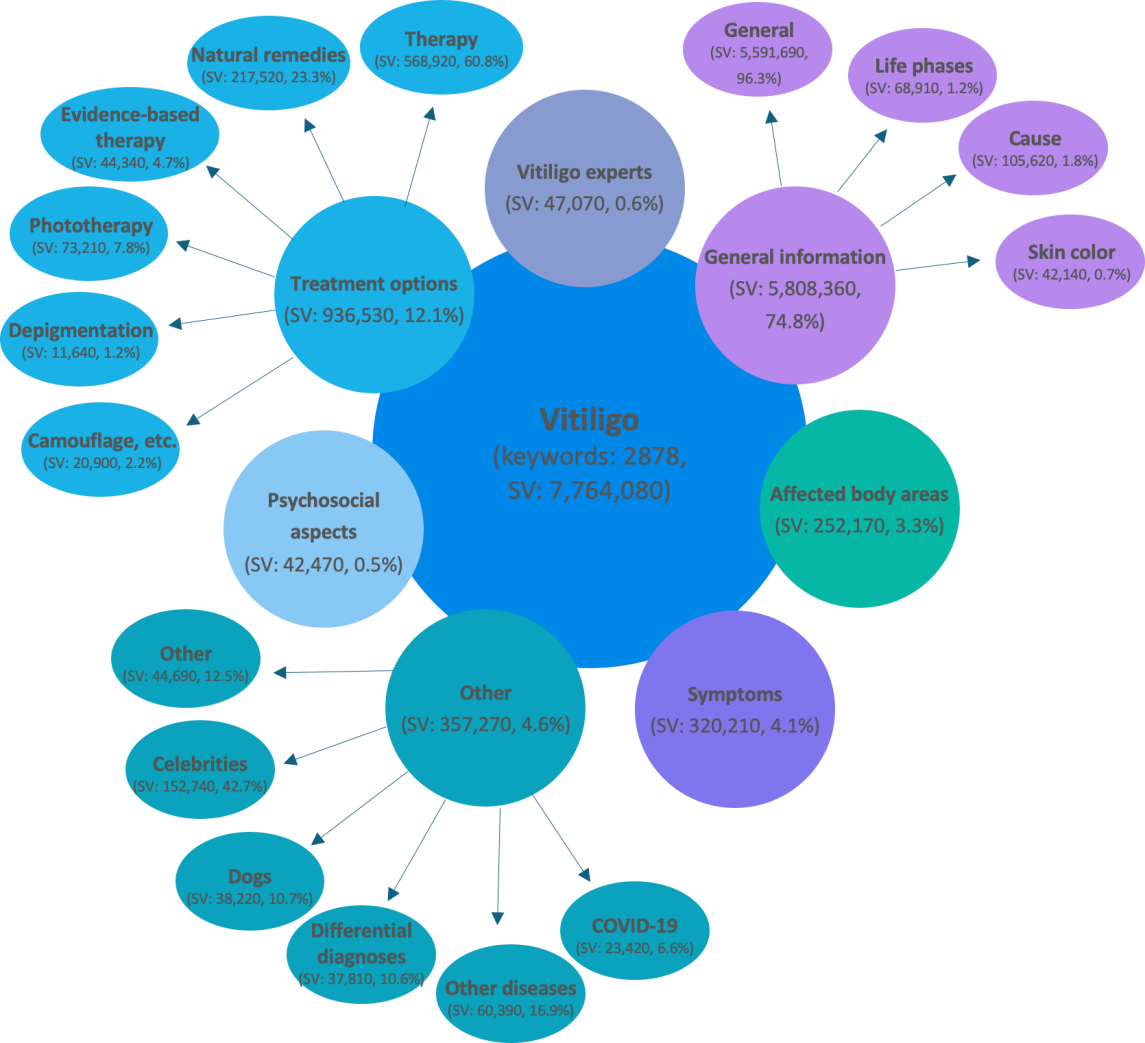
Categorization of the search terms of Google Ads Keyword Planner. SV: search volume.

In the “treatment options” category, 60.75% (568,929/936,530) of the searches referred to general treatment information (therapy). Searches related to “symptoms” accounted for 4.12% (320,210/7,764,080), whereas searches for “affected body areas” made up 3.25% (252,170/7,764,080). Among these, 41.17% (103,820/252,170) focused on the genitoanal region, and 31.76% (80,100/252,170) focused on the face. Searches related to “psychosocial aspects” accounted for only 0.55% (42,470/7,764,080) of all queries. Among Polish-language searches, “psychosocial aspects” represented a markedly higher proportion, accounting for 14.22% (1640/11,530) of all Polish-language queries (Figures2  3).

**Figure 3.**
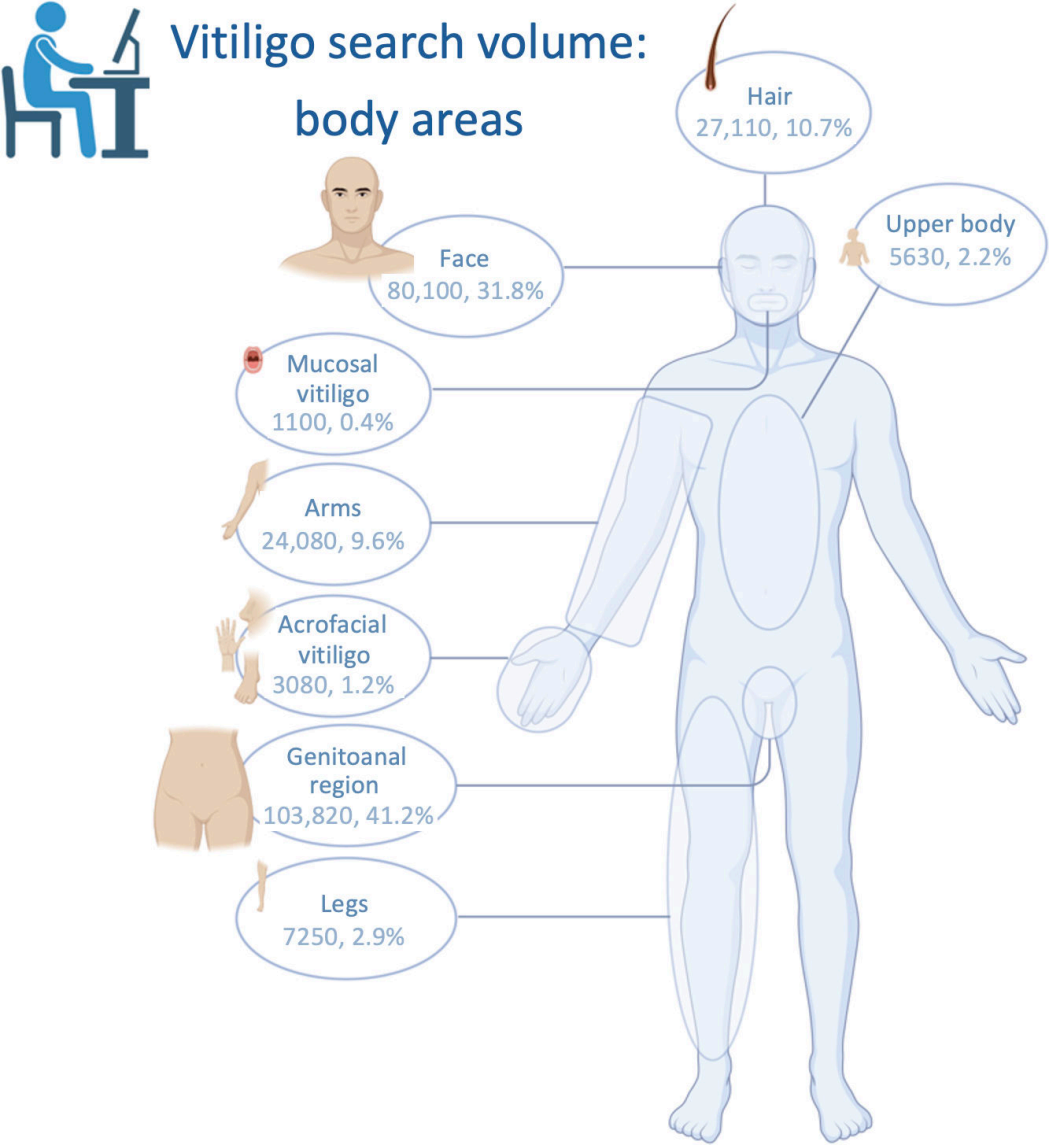
Vitiligo search volume in Germany grouped according to body localizations (created in BioRender).

### Search Volume Over Time

**Figure 4.**
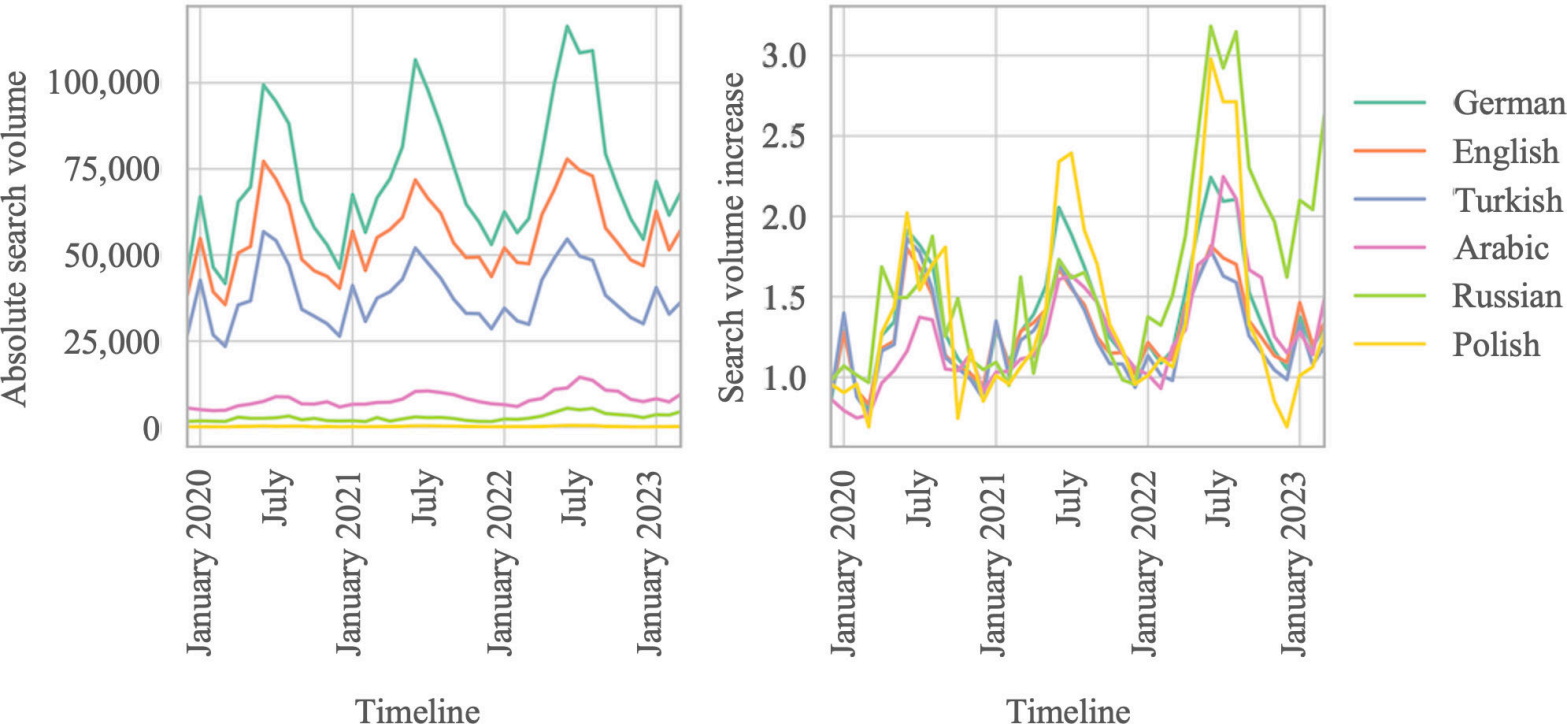
Seasonal search volume in Germany in 6 different languages (C Gasteiger, unpublished data, 2025).

The overall search volume per year increased during the study period. The lowest search volume was recorded in January 2020, and the highest search volume was recorded in June 2022 (Figure 4 [C Gasteiger, unpublished data, 2025]). Seasonal variations in search volume were evident, with significant differences in median search volumes across seasons (*P*<.001), showing peaks in spring and summer. This seasonal effect was observed in all 6 languages (Figure 4).

All languages except Russian showed a steady annual increase in search volume between 2020 and 2022. The search volume in Russian declined by 282,445 searches in 2021 compared to 2020. In 2022, search queries in Russian increased by 653,421 from January onward compared to the previous year.

The seasonality of search volume suggests a possible connection with weather conditions. Sunlight hours (Spearman *R*=0.78; *P*<.001) and temperature (Spearman *R*=0.57; *P*<.001) showed a significant positive correlation with search volume. It is also known that sunlight hours and temperature are strongly positively correlated, reflecting their common seasonal variation. No correlation was found between search volume and precipitation levels (*P*=.28).

The analysis found no correlation between vitiligo-related search volume and COVID-19 or influenza cases in Germany. As expected, a seasonal pattern was observed for pollen levels, with peaks corresponding to the birch pollen season in the spring. The level of birch pollen exposure showed no measurable association with vitiligo-related search volume [34].

### Geographical Distribution of Search Volumes

**Figure 5.**
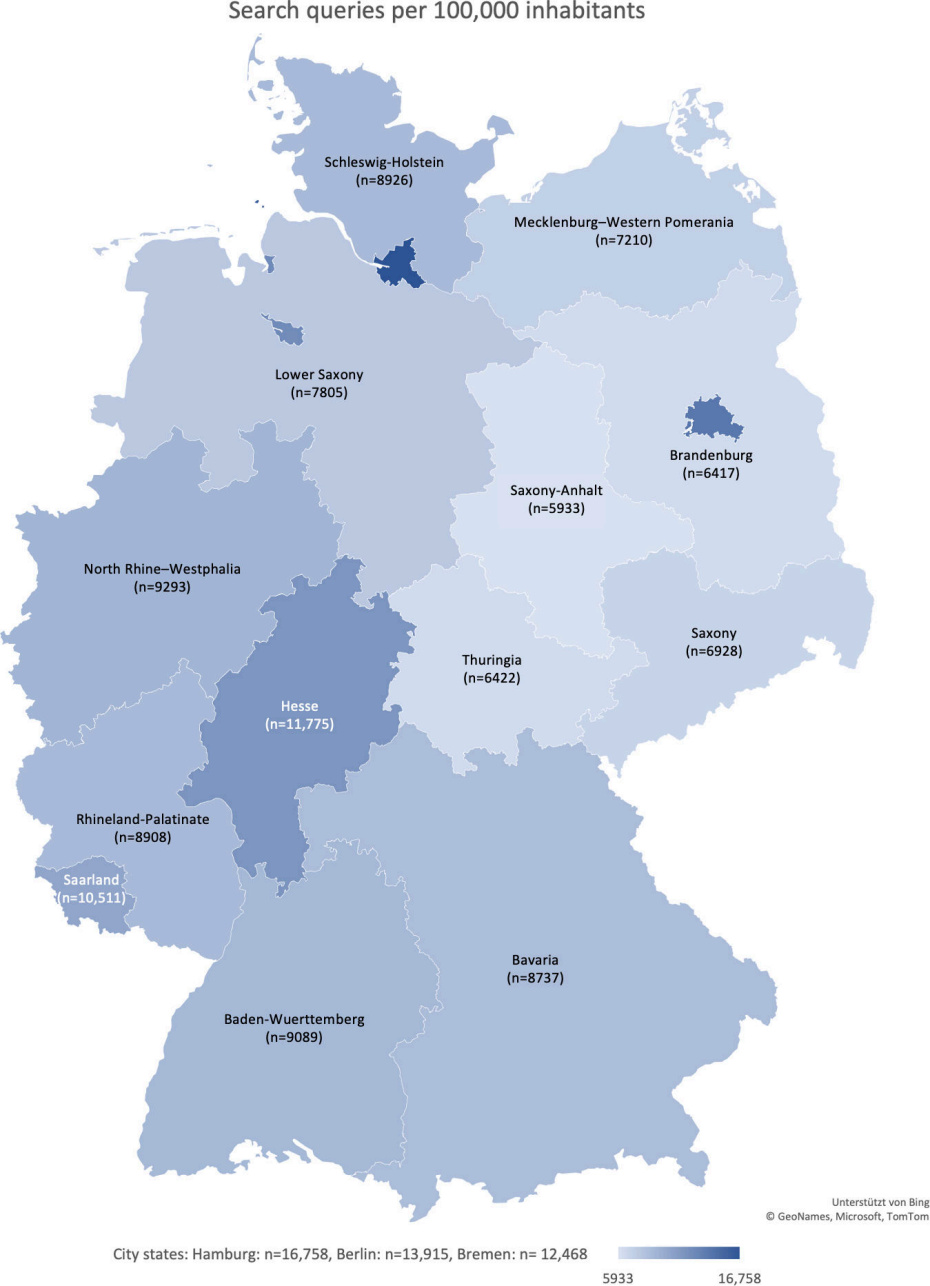
German federal state Google search volume of vitiligo-related terms per 100,000 inhabitants.

Search volumes per 100,000 inhabitants for vitiligo varied substantially across German federal states. The city-states Hamburg (16,758/7,764,080, 0.22%), Berlin (13,915/7,764,080, 0.18%), and Bremen (12,486/7,764,080, 0.16%) recorded the highest search volumes. The lowest search volume per 100,000 inhabitants was found in Saxony-Anhalt (5933/7,764,080, 0.08%; Figure 5).

A significant positive correlation was observed between search volume per 100,000 inhabitants and the population density (*P*<.001), as well as the number of dermatologists per 100,000 inhabitants (*P*<.001). The density of dermatologists was also correlated with population density (*P*<.001). According to the regional typology of the Federal Statistical Office of Germany, search volumes were significantly higher in rural districts than in urban ones (Mann-Whitney *U* test; *P*=.02).

## Discussion

### Principal Findings

This study analyzed public interest in vitiligo using Google search data and identified notable seasonal and regional variations across Germany. Search volume peaked during the summer months and was significantly associated with temperature and sunshine hours. Additionally, rural areas showed higher search volume per 100,000 inhabitants than urban regions. As previous studies have shown, web search analysis is a valuable tool for monitoring disease trends and public interest in health-related topics [35-37]. Given that many patients with skin conditions search online before seeing a physician, these findings offer valuable insights for improving patient education and aligning clinical communication with common concerns.

A total of 7.76 million vitiligo-related searches were recorded between October 2019 and May 2023. Similar international analyses exist, although methodological differences limit comparability [12,13,26]. In line with previous studies on dermatological conditions in Germany [19-22,38,39], the most frequently searched category was “general information.” “Treatment options” ranked second, echoing trends from US data [10] and highlighting persistent demand for effective therapies. Our findings show that public awareness extends beyond general interest in vitiligo and is closely associated with concrete health-related concerns. While general awareness is reflected in the high volume of informational searches, the frequency of treatment- and symptom-related queries indicates an active demand for practical medical guidance. This high search volume focused on general information and treatment options, suggesting considerable uncertainty regarding disease mechanisms and therapeutic approaches. These patterns indicate substantial unmet informational needs, particularly with respect to comorbidities and evidence-based treatment pathways. This aligns with evidence from patient-centered research showing that more than half of individuals with vitiligo report having been told that no adequate treatment exists and a considerable proportion of dermatologists express skepticism regarding the effectiveness of currently available therapeutic options [10]. Dermatologists should recognize this demand and proactively address treatment-related questions during consultations [12]. Notably, “alternative medicine and home remedies” was the second most searched treatment-related subcategory—higher than in US studies [11]—mirroring findings from German psoriasis-related searches [21]. This aligns with a high prevalence of complementary and alternative medicine use in Germany, where up to 40% of individuals report using such therapies annually [40]. Conversely, the psychosocial aspects of vitiligo, though well documented in the literature [5,6,9], accounted for just 0.55% (42,470/7,764,080) of all queries—suggesting a potential disconnect between clinical focus and patient search behavior.

Language distribution revealed that 52.77% (2,428,440/4,601,820) of searches were in English, followed by Turkish, Arabic, Russian, and Polish. This does not reflect native speaker demographics in Germany, where Turkish is the most common non-German language. One explanation could be that health-literate individuals or medical professionals prefer English-language queries when seeking research-based information [41]. Interestingly, Russian-language search volume rose by 65% in 2022, likely related to migration dynamics following the onset of the Russian-Ukrainian war. Between 2022 and 2023, the number of Ukrainian residents in Germany increased 8-fold, whereas Russian nationals rose by 13.7% [30]. The high percentage of treatment-related searches in Arabic may reflect barriers to health care access or lower availability of language-appropriate medical resources. The relatively high interest in psychosocial aspects among Polish searches may point toward culturally mediated coping strategies. These findings underline the importance of multilingual and culturally tailored public health communication strategies [42,43].

Seasonal variation in search interest has been noted in prior global studies [10,11], with increased interest during the summer attributed to greater skin exposure and visibility. Analyses using other tools such as Google Trends likewise revealed seasonal patterns and search term distributions consistent with those in our findings, although these approaches are limited to relative search volumes rather than absolute numbers [12]. Our correlation analyses confirmed strong associations among search volume, temperature, and sunshine hours but not precipitation. Similar seasonal dynamics have been observed in other skin diseases; for example, Tizek et al [19] reported a winter peak in atopic dermatitis searches, which was attributed to seasonal symptom exacerbation.

Regionally, urban areas such as Hamburg, Berlin, and Bremen exhibited the highest search volume per capita, possibly due to younger populations and greater digital health literacy [19,23]. Interestingly, rural areas showed an even higher search volume per 100,000 inhabitants, perhaps reflecting more limited access to dermatologic care and longer wait times—pushing patients to seek digital information first.

These findings can inform public health strategies and clinical practice. Dermatologists and health authorities could develop multilingual digital education materials tailored to commonly searched topics. Seasonal peaks in search activity—particularly in the summer—present opportunities for targeted outreach or awareness campaigns. Addressing the popularity of alternative treatments with evidence-based education may also bridge gaps between public interest and clinical guidance.

### Study Limitations

This study has several limitations. Google’s search volume estimates are derived from a proprietary algorithm that prioritizes frequently searched terms, possibly overrepresenting them. As vitiligo is a rare disease, the absolute search volumes were comparatively low, which may introduce random variation and potential bias in the data interpretation; therefore, the findings should be interpreted with caution, particularly when assessing regional or temporal fluctuations. Moreover, user demographics are not provided, preventing conclusions about specific population segments. As only Google was analyzed, with 95% of the market share in Germany [44], searches in other engines were excluded. As the study period (2019‐2023) largely preceded the widespread use of artificial intelligence (AI)–based search systems (eg, ChatGPT), their impact on search patterns was considered negligible. As AI-driven search tools increasingly shape online information-seeking behavior, future studies should not rely on traditional web search data but adopt integrated approaches that also capture AI-mediated search activity. Internet use also varies by age; although 90% of Germans have internet access [14], older individuals may still be underrepresented. Additionally, web search data can be influenced by repeated queries from the same users or automated activity (bots), which cannot be fully excluded. In the statistical analyses, the assumption of data independence may be partly violated as temporal and spatial correlations can occur in Google search data. While monthly aggregation mitigated short-term autocorrelation, future studies could apply time-series modeling approaches (eg, autoregressive integrated moving average or mixed-effects regression) to better capture dynamic dependencies.

### Study Strengths and Outlook

A major strength of this study is its inclusion of Germany’s 6 most spoken languages, providing a broader, more inclusive picture of public interest. Most previous studies have focused only on English or the dominant national languages. Native speakers validated translated search terms, ensuring linguistic accuracy. Furthermore, the integration of external datasets—on weather, population, and health care access—allowed for robust contextual analysis.

Future research could apply qualitative methods to explore the motivations behind specific search behaviors and investigate how this correlates with clinical factors such as disease severity or treatment history. Analyzing social media platforms may also offer insights into community-level discussions and shared experiences related to vitiligo.

### Conclusions

This study provides valuable insights into public interest in vitiligo through an analysis of Google search engine data in Germany. To promote inclusivity and representativeness, the 6 most widely spoken languages were included. As most of the searches focused on general information and general treatment options, this study suggests that, in addition to individuals diagnosed with vitiligo, many people may seek information online before consulting a physician. This highlights the potential to improve disease education by providing accessible and reliable online resources, such as government-funded websites with evidence-based information on vitiligo and treatment options, and the need for multilingual and culturally sensitive health information resources that acknowledge different approaches to disease management. The observed fluctuations in search activity may reflect variations in public awareness, perceived disease burden, and information-seeking behavior among individuals affected by or concerned about vitiligo. Higher search volumes could indicate periods of increased psychosocial stress, symptom exacerbation, or heightened media attention, whereas lower activity might correspond to times of reduced public discourse. Thus, such patterns can serve as a digital proxy for health-related interest and unmet informational needs in the general population. Understanding these dynamics may help clinicians and public health authorities identify temporal windows for targeted awareness campaigns and patient education. In general, monitoring online search trends can help evaluate the effectiveness of awareness campaigns and identify gaps in public knowledge. Clinical outcomes and qualitative research could further enhance our understanding of how online health information–seeking behavior relates to patient care and disease management.

## Abbreviations

AI: artificial intelligence

## References

[1] Böhm M, Schunter JA, Fritz K (2022). S1 guideline: diagnosis and therapy of vitiligo. J Dtsch Dermatol Ges.

[2] Ezzedine K, Eleftheriadou V, Whitton M, van Geel N (2015). Vitiligo. Lancet.

[3] Alikhan A, Felsten LM, Daly M, Petronic-Rosic V (2011). Vitiligo: a comprehensive overview part I. Introduction, epidemiology, quality of life, diagnosis, differential diagnosis, associations, histopathology, etiology, and work-up. J Am Acad Dermatol.

[4] Lee JH, Ju HJ, Seo JM (2023). Comorbidities in patients with vitiligo: a systematic review and meta-analysis. J Invest Dermatol.

[5] Lai YC, Yew YW, Kennedy C, Schwartz RA (2017). Vitiligo and depression: a systematic review and meta-analysis of observational studies. Br J Dermatol.

[6] Bibeau K, Ezzedine K, Harris JE (2023). Mental health and psychosocial quality-of-life burden among patients with vitiligo: findings from the global VALIANT study. JAMA Dermatol.

[7] Cunningham KN, Rosmarin D (2023). Vitiligo treatments: review of current therapeutic modalities and JAK inhibitors. Am J Clin Dermatol.

[8] Seneschal J, Speeckaert R, Taïeb A (2023). Worldwide expert recommendations for the diagnosis and management of vitiligo: position statement from the international vitiligo task force-part 2: specific treatment recommendations. J Eur Acad Dermatol Venereol.

[9] Eleftheriadou V, Delattre C, Chetty-Mhlanga S (2024). Burden of disease and treatment patterns in patients with vitiligo: findings from a national longitudinal retrospective study in the UK. Br J Dermatol.

[10] Hamzavi IH, Bibeau K, Grimes P (2023). Exploring the natural and treatment history of vitiligo: perceptions of patients and healthcare professionals from the global VALIANT study. Br J Dermatol.

[11] Ezzedine K, Eleftheriadou V, Jones H (2021). Psychosocial effects of vitiligo: a systematic literature review. Am J Clin Dermatol.

[12] Montgomery SN, Elbuluk N (2020). Evaluating population interest in vitiligo through an analysis of google trends and social media. J Drugs Dermatol.

[13] Speeckaert R, Van Geel N (2021). What vitiligo patients want to know outside the dermatologist’s office: an analysis of online search behaviour. Eur J Dermatol.

[14] van Geel N, Speeckaert R, Taïeb A (2023). Worldwide expert recommendations for the diagnosis and management of vitiligo: position statement from the International Vitiligo Task Force part 1: towards a new management algorithm. J Eur Acad Dermatol Venereol.

[15] Ghafourian A, Ghafourian S, Sadeghifard N (2014). Vitiligo: symptoms, pathogenesis and treatment. Int J Immunopathol Pharmacol.

[16] (2024). ARD/ZDF-medienstudie 2024. https://www.ard-zdf-medienstudie.de.

[17] (2019). Mobile search engine market share Germany (oct 2019 - may 2023). Statcounter.

[18] Kain A, Tizek L, Wecker H, Wallnöfer F, Biedermann T, Zink A (2023). Evaluating public interest in herpes zoster in Germany by leveraging the internet: a retrospective search data analysis. BMC Public Health.

[19] Tizek L, Schielein MC, Tizek L, Zink A (2022). Atopic dermatitis-identifying needs in the German population by internet search queries. Hautarzt.

[20] Schober A, Tizek L, Johansson EK (2022). Monitoring disease activity of pollen allergies: what crowdsourced data are telling us. World Allergy Organ J.

[21] Wallnöfer F, Erbas ME, Tizek L (2022). Leveraging web search data to explore public interest in psoriasis in Germany. JEADV Clin Pract.

[22] Wecker H, Maier D, Ziehfreund S (2024). Cancer incidence and digital information seeking in Germany: a retrospective observational study. Sci Rep.

[23] Wecker H, Ziehfreund S, Hindelang M, Welcker M, Zink A (2024). Change of perspective: impact of COVID-19 pandemic on axial spondyloarthritis-related web searches in Germany. Sci Rep.

[24] Mick A, Wecker H, Ziehfreund S, Maul JT, Biedermann T, Zink A (2024). Cracking the code: unveiling the nexus between atopic dermatitis and addictive behavior: a cross-sectional exploration of risk factors. Arch Dermatol Res.

[25] Berr K, Tizek L, Schielein MC (2023). Analyzing web searches for axial spondyloarthritis in Germany: a novel approach to exploring interests and unmet needs. Rheumatol Int.

[26] Kluger N (2019). The Michael Jackson and Winnie Harlow effect: impact on vitiligo awareness on the internet. J Clin Aesthet Dermatol.

[27] Pilz AC, Tizek L, Rüth M, Seiringer P, Biedermann T, Zink A (2021). Interest in sexually transmitted infections: analysis of web search data terms in eleven large German cities from 2015 to 2019. Int J Environ Res Public Health.

[28] Tizek L, Schielein MC, Rüth M (2019). Interest in skin cancer in urban populations: a retrospective analysis of Google search terms in nine large German cities. Acta Derm Venereol.

[29] Scheerer C, Rüth M, Tizek L, Köberle M, Biedermann T, Zink A (2020). Googling for ticks and borreliosis in Germany: nationwide google search analysis from 2015 to 2018. J Med Internet Res.

[30] (2022). Bevölkerung nach nationalität und bundesländern. Statistisches Bundesamt (Destatis).

[31] (2023). Indikatoren und karten zur raum- und stadtentwicklung. INKAR.

[32] Regionale verteilung der ärztinnen und ärzte in der vertragsärztlichen versorgung. Kassenärztliche Bundesvereinigung (KBV).

[33] Daily dataset of 20th-century surface air temperature and precipitation series for the European climate assessment. European Climate Assessment & Dataset.

[34] van den Besselaar EJ, Sanchez-Lorenzo A, Wild M, Klein Tank AM, de Laat AT (2015). Relationship between sunshine duration and temperature trends across Europe since the second half of the twentieth century. J Geophys Res Atmos.

[35] Seth D, Gittleman H, Barnholtz-Sloan J, Bordeaux JS (2018). Association of socioeconomic and geographic factors with Google trends for tanning and sunscreen. Dermatol Surg.

[36] Amante DJ, Hogan TP, Pagoto SL, English TM, Lapane KL (2015). Access to care and use of the Internet to search for health information: results from the US National Health Interview survey. J Med Internet Res.

[37] Zink A, Schuster B, Rüth M (2019). Medical needs and major complaints related to pruritus in Germany: a 4-year retrospective analysis using Google AdWords keyword planner. J Eur Acad Dermatol Venereol.

[38] Hilker C, Tizek L, Rüth M, Schielein M, Biedermann T, Zink A (2021). Leveraging internet search data to assess prevalence, interest, and unmet needs of sarcoidosis in Germany. Sci Rep.

[39] Mick A, Tizek L, Schielein M, Zink A (2022). Can crowdsourced data help to optimize atopic dermatitis treatment? Comparing web search data and environmental data in Germany. J Eur Acad Dermatol Venereol.

[40] Kemppainen LM, Kemppainen TT, Reippainen JA, Salmenniemi ST, Vuolanto PH (2018). Use of complementary and alternative medicine in Europe: Health-related and sociodemographic determinants. Scand J Public Health.

[41] (2024). 80% der bevölkerung sprechen zu hause ausschließlich deutsch. Statistisches Bundesamt (Destatis).

[42] Narla S, Heath CR, Alexis A, Silverberg JI (2023). Racial disparities in dermatology. Arch Dermatol Res.

[43] Thein K, Erim Y, Morawa E (2020). Comparison of illness concepts and coping strategies among cancer patients of Turkish and German origin. Int J Environ Res Public Health.

[44] (2023). Mobile search engine market share in Germany - December 2023. Statcounter.

